# Supplementary material for: Contamination Status of Pet Cats in Thailand with Organohalogen Compounds (OHCs) and Their Hydroxylated and Methoxylated Derivatives and Estimation of Sources of Exposure to These Contaminants
Source: Animals (Basel). 2022 Dec 13;12(24):3520. doi: 10.3390/ani12243520 (PMC9774237; doi:10.3390/ani12243520)
Supplement: Supplementary file 1 [file animals-12-03520-s001.zip › animals-2048823-supplementary.pdf]

**Supplementary materials**

# Contamination status of pet cats in Thailand with organohalogen compounds (OHCs) and their hydroxylated and methoxylated derivatives and estimation of sources of exposure to these contaminants

**Makoto Shimasaki[1], Hazuki Mizukawa[1,2], Kohki Takaguchi[3], Aksorn Saengtienchai[4], Araya Ngamchirttakul[5], Disdanai Pencharee[6], Kraisiri Khidkhan[4,7], Yoshinori Ikenaka[7,8,9], Shouta M.M. Nakayama[7], Mayumi Ishizuka[7], and Kei Nomiyama[1,*]**

[1] Center for Marine Environmental Studies (CMES), Ehime University, Bunkyo-cho 2-5, Matsuyama, Ehime 790-8577, Japan
[2] Department of Science and Technology for Biological Resources and Environment, Graduate School of Agriculture, Ehime University, Tarumi 3-5-7, Matsuyama, Ehime, 790-8566 Japan
[3] Center for Preventive Medical Sciences, Chiba University, 6-2-1 Kashiwanoha, Kashiwa, Chiba 277-0882, Japan
[4] Department of Pharmacology, Faculty of Veterinary Medicine, Kasetsart University, 50 Ngam Wong Wan Rd, Lat Yao, Chatuchak, Bangkok 10900, Thailand
[5] Kasetsart University Veterinary Teaching Hospital Nong Pho, Faculty of Veterinary Medicine, Kasetsart University, Ratchaburi, Thailand.
[6] Kasetsart University Veterinary Teaching Hospital Hua Hin, Faculty of Veterinary Medicine, Kasetsart University, Prachuap Khiri Khan, Thailand.
[7] Department of Environmental Veterinary Sciences, Faculty of Veterinary Medicine, Hokkaido University, Kita 18, Nishi 9, Kita-ku, Sapporo, Hokkaido 060-0818, Japan
[8] Veterinary Teaching Hospital, Graduate School of Veterinary Medicine, Hokkaido University, N18 W9, Sapporo, Hokkaido 060-0818, Japan
[9] One Health Research Center, Hokkaido University, Kita 18, Nishi 9, Kita-ku, Sapporo 060-0818, Japa
***** Correspondence: nomiyama.kei.mb@ehime-u.ac.jp; Tel.: +81-(089)-927-8196

*Corresponding author: Kei Nomiyama, Ph.D.

E-mail: nomiyama.kei.mb@ehime-u.ac.jp

- **Table S1.** Results on questionnaire for house dust sampling. **P3.**

- **Table S2.** Concentrations of PBDEs in cat sera [pg/mL], dry and wet cat food [pg/g wet wt.] and house dust [pg $g^{-1}$ dry wt.]. **P4.**

- **Table S3.** Concentrations of PCBs in cat sera [pg/mL], dry and wet cat food [pg/g wet wt.] and house dust [pg $g^{-1}$ dry wt.]. **P5-6.**

- **Table S4.** Concentrations of OH-PCBs in cat sera [pg/mL], dry and wet cat food [pg/g wet wt.] and house dust [pg $g^{-1}$ dry wt.]. **P7-8.**

- **Table S5**. Concentrations of OH-PBDEs in cat sera [pg/mL], dry and wet cat food [pg/g wet wt.] and house dust [pg $g^{-1}$ dry wt.]. **P9.**

- **Table S6.** Concentrations of MeO-PBDEs in cat sera [pg/mL], dry and wet cat food [pg/g wet wt.] and house dust [pg $g^{-1}$ dry wt.]. **P10.**

- **Table S7.** Lipid-based concentrations (ng $g^{-1}$) of OHCs in cat serum ($n$ = 26). **P11.**

- **Table S8**. Correlations between the concentrations [wet wt. base] of OHCs in cat sera and the results of questionnaires. **P12.**

- **Table S9**. Correlations between the concentrations [lipid wt. base] of OHCs in cat sera and the results of questionnaires*. **P13.**

- **Table S10.** Estimation of daily intake of BDE-47 via cat food and house dust. **P14.**

- **Table. S11.** Estimation of daily intake of BDE-99 via cat food and house dust. **P15.**

- **Table S12.** Estimation of daily intake of BDE-153 via cat food and house dust. **P16.**

- **Table S13.** Estimation of daily intake of BDE-209 via cat food and house dust. **P17.**

**Table S1.** Results on questionnaire for house dust sampling.

| Sample name | Room | Area/m$^2$ | Number of cleanings per week* | Electric appliance & Furniture | | | | | | | | | | | | |
|---|---|---|---|---|---|---|---|---|---|---|---|---|---|---|---|---|
| | | | | Flooring | Number of window | Ventilation frequency | Number of TV | Number of desktop PC | Number of air conditioner | Other electric appliances | Other electric appliancws | Number of sofa | Number of curtain | Number of Bed | Other furniture | Other furniture |
| TH-DUST-1 | Living | 3×3 | 3 | Wooden floor | 16 | all times | 1 | - | - | Fan (3) | | 2 | - | - | | - |
| | Dining room | 3×4 | 3 | Stone | 5 | all times | - | - | - | Pot (1) | Rice cooker (1) | - | - | - | | - |
| | Bed room | 3×3 | 3 | Wooden floor | 8 | 2 times / day | - | - | - | Radio (1) | | - | 2 | - | | - |
| TH-DUST-4 | Living | 10×10 | 2 | Stone | 10 | all times | 1 | - | - | Freezer (1) | - | - | - | - | Wood table (1) | Wood chair (6) |
| | Dining room | 3×4 | 3 | Stone | 2 | all times | - | - | - | Microwave oven (1), Cooker (1) | Pot (1) | - | - | - | Wood table (1) | Wood chair (4) |
| | Bed room | 5×6 | 1 | Stone | 4 | all times | 1 | 2 | 1 | Fan (2) | - | - | - | 1 | - | - |
| TH-DUST-5 | Living | 3×3 | 2 | Stone | 6 | all times | 1 | - | - | - | - | - | - | 1 | - | - |
| | Dining room | 2×3 | 2 | Stone | 4 | all times | - | - | - | Pot (1), Oven (1) | Freezer (1) | - | - | - | Wood table (1) | Wood chair (2) |
| | Bed room | 3×3 | 2 | Wooden floor | 8 | all times | - | - | - | - | Fan (3) | - | - | 1 | - | - |
| TH-DUST-6 | Living | 10×5 | 1 | Stone | Open | all times | 1 | 1 | - | Fan (1) | - | - | - | - | Wood table (1) | Wood chair (5) |
| | Dining room | 3×3 | 1 | Stone | Open | all times | - | - | - | Pot (1), Freezer (1) | Cooker (1) | - | - | - | Wood table (2) | Wood chair (8) |
| | Bed room | 3.5×3.5 | 1 | Wooden floor | 8 | all times | - | - | - | Fan (1) | - | - | - | 1 | - | - |
| TH-DUST-7 | Living | 6×6 | 2 | Wooden floor | Open | all times | 1 | - | - | Fan (2) | - | - | - | - | Wood table (2) | Wood chair (10) |
| | Dining room | 4×4 | 2 | Stone | 6 | 2 | - | - | - | Cooker (1), Pot (1) | Oven (1), Freezer (1) | - | - | - | Wood table (1) | Wood chair (5) |
| | Bed room | 3×3 | 2 | Wooden floor | 4 | 1 | 1 | - | 1 | Fan (1) | - | - | - | 1 | - | - |
| TH-DUST-8 | Living | 2×2 | 3 | Stone | 3 | 1 | 1 | - | - | Fan (1) | - | 1 | 3 | - | Wood table (1) | - |
| | Dining room | 3×3 | 3 | Stone | 3 | 1 | - | - | - | Pot (1) | Cooker (1) | - | - | - | Wood table (1) | - |
| | Bed room | 2×3 | 1 | Stone | 3 | 1 | - | - | - | Fan (1) | - | - | 3 | 1 | Wood wardrobe (1) | - |
| TH-DUST-13 | Living | 3×4 | 2 | Wooden floor | 2 | 1 | - | 1 | 1 | - | - | - | - | - | Wood table (1) | Wood chair (1) |
| | Dining room | 3×3 | 2 | Wooden floor | 3 | 1 | - | - | - | Pot (1) | - | - | - | - | - | - |
| | Bed room | 2×3 | 2 | Wooden floor | 1 | 1 | - | - | - | Fan (1) | - | - | 1 | 1 | - | - |

**Table S2.** Concentrations of PBDEs in cat sera [pg mL$^{-1}$], dry and wet cat food [pg g$^{-1}$ wet wt.] and house dust [pg g$^{-1}$ dry wt.].

| | Cat sera | | | | | | Dry food | | | | | | Wet food | | | | | | House dust | | | | | |
|---|---|---|---|---|---|---|---|---|---|---|---|---|---|---|---|---|---|---|---|---|---|---|---|---|
| **PBDEs** | Mean | SD | Median | Range | DF [%] | MDL | Mean | SD | Median | Range | DF [%] | MDL | Mean | SD | Median | Range | DF [%] | MDL | Mean | SD | Median | Range | DF [%] | MDL |
| **BDE47** | 4.2 | 11 | <MDL | <MDL - 70 | 26 | 6.1 | 5.0 | 5.1 | 4.9 | <MDL - 15 | 71 | 2.1 | 11 | 16 | 5.6 | <MDL - 46 | 71 | 2.1 | 320 | 270 | 260 | 110 - 890 | 100 | 110 |
| **BDE49** | <MDL | - | <MDL | <MDL | 0 | 8.0 | 1.1 | 2.9 | <MDL | <MDL - 7.7 | 14 | 3.3 | <MDL | - | <MDL | <MDL | 0 | 3.3 | <MDL | - | <MDL | <MDL | 0 | 110 |
| **BDE71** | <MDL | - | <MDL | <MDL | 0 | 9.7 | <MDL | - | <MDL | <MDL | 0 | 4.2 | 5.9 | 16 | <MDL | <MDL - 41 | 14 | 4.2 | <MDL | - | <MDL | <MDL | 0 | 200 |
| **BDE66** | <MDL | - | <MDL | <MDL | 0 | 13 | <MDL | - | <MDL | <MDL | 0 | 6.8 | <MDL | - | <MDL | <MDL | 0 | 6.8 | <MDL | - | <MDL | <MDL | 0 | 130 |
| **BDE77** | <MDL | - | <MDL | <MDL | 0 | 13 | <MDL | - | <MDL | <MDL | 0 | 3.9 | <MDL | - | <MDL | <MDL | 0 | 3.9 | <MDL | - | <MDL | <MDL | 0 | 290 |
| **BDE100** | 0.25 | 1.6 | <MDL | <MDL - 10 | 2.6 | 8.6 | 1.1 | 1.8 | <MDL | <MDL - 3.9 | 29 | 3.1 | 4.7 | 9.1 | <MDL | <MDL - 25 | 43 | 3.1 | 41 | 110 | <MDL | <MDL - 290 | 14 | 150 |
| **BDE99** | 5.4 | 16 | <MDL | <MDL - 95 | 21 | 11 | 2.8 | 4.9 | <MDL | <MDL - 12 | 29 | 4.4 | 1.6 | 4.3 | <MDL | <MDL - 11 | 14 | 4.4 | 410 | 450 | 210 | 130 - 1400 | 100 | 120 |
| **BDE119** | <MDL | - | <MDL | <MDL | 0 | 17 | <MDL | - | <MDL | <MDL | 0 | 2.6 | <MDL | - | <MDL | <MDL | 0 | 2.6 | <MDL | - | <MDL | <MDL | 0 | 200 |
| **BDE85** | <MDL | - | <MDL | <MDL | 0 | 16 | <MDL | - | <MDL | <MDL | 0 | 5.0 | <MDL | - | <MDL | <MDL | 0 | 5.0 | <MDL | - | <MDL | <MDL | 0 | 100 |
| **BDE126** | <MDL | - | <MDL | <MDL | 0 | 18 | <MDL | - | <MDL | <MDL | 0 | 6.5 | <MDL | - | <MDL | <MDL | 0 | 6.5 | <MDL | - | <MDL | <MDL | 0 | 200 |
| **BDE154** | 0.53 | 3.4 | <MDL | <MDL - 22 | 2.6 | 17 | <MDL | - | <MDL | <MDL | 0 | 4.6 | <MDL | - | <MDL | <MDL | 0 | 4.6 | <MDL | - | <MDL | <MDL | 0 | 150 |
| **BDE153** | 3.9 | 18 | <MDL | <MDL - 110 | 5.1 | 16 | <MDL | - | <MDL | <MDL | 0 | 2.4 | <MDL | - | <MDL | <MDL | 0 | 2.4 | 62 | 160 | <MDL | <MDL - 430 | 14 | 390 |
| **BDE139** | <MDL | - | <MDL | <MDL | 0 | 17 | <MDL | - | <MDL | <MDL | 0 | 7.5 | <MDL | - | <MDL | <MDL | 0 | 7.5 | <MDL | - | <MDL | <MDL | 0 | 290 |
| **BDE140** | <MDL | - | <MDL | <MDL | 0 | 23 | <MDL | - | <MDL | <MDL | 0 | 4.2 | <MDL | - | <MDL | <MDL | 0 | 4.2 | <MDL | - | <MDL | <MDL | 0 | 220 |
| **BDE138** | <MDL | - | <MDL | <MDL | 0 | 43 | <MDL | - | <MDL | <MDL | 0 | 15 | <MDL | - | <MDL | <MDL | 0 | 15 | <MDL | - | <MDL | <MDL | 0 | 690 |
| **BDE169** | <MDL | - | <MDL | <MDL | 0 | 43 | <MDL | - | <MDL | <MDL | 0 | 4.4 | <MDL | - | <MDL | <MDL | 0 | 4.4 | <MDL | - | <MDL | <MDL | 0 | 690 |
| **BDE183** | 11 | 61 | <MDL | <MDL - 390 | 5.1 | 18 | 9.0 | 9.7 | 12 | <MDL - 26 | 57 | 8.9 | <MDL | - | <MDL | <MDL | 0 | 8.9 | 480 | 490 | 640 | <MDL - 1300 | 57 | 170 |
| **BDE184** | <MDL | - | <MDL | <MDL | 0 | 18 | <MDL | - | <MDL | <MDL | 0 | 6.8 | <MDL | - | <MDL | <MDL | 0 | 6.8 | <MDL | - | <MDL | <MDL | 0 | 530 |
| **BDE180** | <MDL | - | <MDL | <MDL | 0 | 39 | <MDL | - | <MDL | <MDL | 0 | 11 | <MDL | - | <MDL | <MDL | 0 | 11 | <MDL | - | <MDL | <MDL | 0 | 550 |
| **BDE171** | <MDL | - | <MDL | <MDL | 0 | 19 | <MDL | - | <MDL | <MDL | 0 | 5.2 | <MDL | - | <MDL | <MDL | 0 | 5.2 | <MDL | - | <MDL | <MDL | 0 | 820 |
| **BDE191** | <MDL | - | <MDL | <MDL | 0 | 30 | <MDL | - | <MDL | <MDL | 0 | 22 | <MDL | - | <MDL | <MDL | 0 | 22 | <MDL | - | <MDL | <MDL | 0 | 670 |
| **BDE197+204** | 11 | 27 | <MDL | <MDL - 130 | 31 | 8.4 | 9.6 | 21 | <MDL | <MDL - 57 | 29 | 5.2 | <MDL | - | <MDL | <MDL | 0 | 5.2 | 290 | 240 | 350 | <MDL - 650 | 71 | 86 |
| **BDE196** | 9.2 | 31 | <MDL | <MDL - 170 | 13 | 26 | 19 | 50 | <MDL | <MDL - 130 | 14 | 17 | <MDL | - | <MDL | <MDL | 0 | 17 | 710 | 590 | 890 | <MDL - 1400 | 71 | 270 |
| **BDE201** | 17 | 38 | <MDL | <MDL - 150 | 46 | 8.7 | 11 | 28 | <MDL | <MDL - 75 | 14 | 13 | <MDL | - | <MDL | <MDL | 0 | 13 | 300 | 420 | <MDL | <MDL - 1100 | 43 | 430 |
| **BDE203** | 12 | 48 | <MDL | <MDL - 270 | 10 | 23 | 34 | 89 | <MDL | <MDL - 240 | 14 | 18 | <MDL | - | <MDL | <MDL | 0 | 18 | 930 | 980 | 1000 | <MDL - 2500 | 57 | 610 |
| **BDE205** | 0.82 | 5.2 | <MDL | <MDL - 33 | 2.6 | 29 | <MDL | - | <MDL | <MDL | 0 | 6.7 | <MDL | - | <MDL | <MDL | 0 | 6.7 | <MDL | - | <MDL | <MDL | 0 | 460 |
| **BDE206** | 90 | 370 | <MDL | <MDL - 2000 | 13 | 66 | 200 | 530 | <MDL | <MDL - 1400 | 14 | 25 | <MDL | - | <MDL | <MDL | 0 | 25 | 15000 | 14000 | 14000 | <MDL - 40000 | 86 | 2000 |
| **BDE207** | 250 | 810 | 74 | <MDL - 4500 | 79 | 36 | 250 | 650 | <MDL | <MDL - 1700 | 14 | 30 | <MDL | - | <MDL | <MDL | 0 | 30 | 11000 | 7000 | 10000 | 2000 - 22000 | 100 | 680 |
| **BDE208** | 180 | 630 | <MDL | <MDL - 3900 | 44 | 47 | 160 | 430 | <MDL | <MDL - 1100 | 14 | 24 | <MDL | - | <MDL | <MDL | 0 | 24 | 5400 | 3900 | 6600 | <MDL - 11000 | 86 | 1600 |
| **BDE209** | 1900 | 6800 | 350 | <MDL - 37000 | 79 | 67 | 4700 | 12000 | 330 | 180 - 31000 | 100 | 15 | 19 | 33 | <MDL | <MDL - 69 | 29 | 15 | 240000 | 200000 | 200000 | 21000 - 520000 | 100 | 5600 |

| | | | | | | | | | | | | | | | | | | | | | | | | |
|---|---|---|---|---|---|---|---|---|---|---|---|---|---|---|---|---|---|---|---|---|---|---|---|---|
| **ΣPBDEs** | 2500 | 8600 | 490 | <MDL - 48000 | 100 | | 5400 | 13000 | 350 | 200 - 36000 | 100 | | 42 | 70 | 12 | <MDL - 190 | 71 | | 270000 | 230000 | 240000 | 26000 - 590000 | 100 | |

**Table S3.** Concentrations of PCBs in cat sera [pg mL$^{-1}$], dry and wet cat food [pg g$^{-1}$ wet wt.] and house dust [pg g$^{-1}$ dry wt.].

| | Cat sera | | | | | | Dry food | | | | | | Wet food | | | | | | House dust | | | | | |
|---|---|---|---|---|---|---|---|---|---|---|---|---|---|---|---|---|---|---|---|---|---|---|---|---|
| **PCBs** | Mean | SD | Median | Range | DF (%) | MDL | Mean | SD | Median | Range | DF (%) | MDL | Mean | SD | Median | Range | DF (%) | MDL | Mean | SD | Median | Range | DF (%) | MDL |
| **CB-19** | <MDL | - | <MDL | <MDL | 0 | 9.0 | <MDL | - | <MDL | <MDL | 0 | 4.1 | <MDL | - | <MDL | <MDL | 0 | 4.1 | <MDL | - | <MDL | <MDL | 0 | 440 |
| **CB-18** | <MDL | - | <MDL | <MDL | 0 | 13 | 2.5 | 6.5 | <MDL | <MDL - 17 | 14 | 6.1 | 4.8 | 13 | <MDL | <MDL - 33 | 14 | 6.1 | <MDL | - | <MDL | <MDL | 0 | 300 |
| **CB-28** | 1.2 | 3.9 | <MDL | <MDL - 17 | 10 | 9.2 | 19 | 21 | 14 | <MDL - 56 | 57 | 8.3 | 7.6 | 20 | <MDL | <MDL - 53 | 14 | 8.3 | <MDL | - | <MDL | <MDL | 0 | 320 |
| **CB-33** | <MDL | - | <MDL | <MDL | 0 | 9.4 | 4.5 | 8.0 | <MDL | <MDL - 20 | 29 | 3.4 | 2.4 | 6.5 | <MDL | <MDL - 17 | 14 | 3.4 | <MDL | - | <MDL | <MDL | 0 | 170 |
| **CB-22** | <MDL | - | <MDL | <MDL | 0 | 8.7 | <MDL | - | <MDL | <MDL | 0 | 5.3 | <MDL | - | <MDL | <MDL | 0 | 5.3 | <MDL | - | <MDL | <MDL | 0 | 190 |
| **CB-37** | <MDL | - | <MDL | <MDL | 0 | 13 | <MDL | - | <MDL | <MDL | 0 | 9.3 | <MDL | - | <MDL | <MDL | 0 | 9.3 | <MDL | - | <MDL | <MDL | 0 | 290 |
| **CB-54** | <MDL | - | <MDL | <MDL | 0 | 7.1 | <MDL | - | <MDL | <MDL | 0 | 4.4 | <MDL | - | <MDL | <MDL | 0 | 4.4 | <MDL | - | <MDL | <MDL | 0 | 250 |
| **CB-52** | 0.57 | 2.6 | <MDL | <MDL - 12 | 5.1 | 9.5 | <MDL | - | <MDL | <MDL | 0 | 7.9 | 7.3 | 19 | <MDL | <MDL - 51 | 14 | 7.9 | 29 | 76 | <MDL | <MDL - 200 | 14 | 170 |
| **CB-49** | <MDL | - | <MDL | <MDL | 0 | 12 | <MDL | - | <MDL | <MDL | 0 | 8.8 | 3.6 | 10 | <MDL | <MDL - 25 | 14 | 8.8 | <MDL | - | <MDL | <MDL | 0 | 140 |
| **CB-44** | <MDL | - | <MDL | <MDL | 0 | 9.7 | <MDL | - | <MDL | <MDL | 0 | 5.4 | <MDL | - | <MDL | <MDL | 0 | 5.4 | <MDL | - | <MDL | <MDL | 0 | 210 |
| **CB-74** | 0.28 | 1.8 | <MDL | <MDL - 11 | 2.6 | 7.4 | 1.5 | 4.0 | <MDL | <MDL - 11 | 14 | 6.4 | 4.2 | 11 | <MDL | <MDL - 29 | 14 | 6.4 | <MDL | - | <MDL | <MDL | 0 | 150 |
| **CB-70** | 0.24 | 1.6 | <MDL | <MDL - 10 | 2.6 | 4.6 | 1.6 | 4.1 | <MDL | <MDL - 11 | 14 | 6.0 | 7.4 | 20 | <MDL | <MDL - 52 | 14 | 6.0 | <MDL | - | <MDL | <MDL | 0 | 330 |
| **CB-81** | <MDL | - | <MDL | <MDL | 0 | 4.5 | <MDL | - | <MDL | <MDL | 0 | 4.2 | <MDL | - | <MDL | <MDL | 0 | 4.2 | <MDL | - | <MDL | <MDL | 0 | 220 |
| **CB-77** | <MDL | - | <MDL | <MDL | 0 | 5.9 | <MDL | - | <MDL | <MDL | 0 | 3.8 | <MDL | - | <MDL | <MDL | 0 | 3.8 | <MDL | - | <MDL | <MDL | 0 | 230 |
| **CB-104** | <MDL | - | <MDL | <MDL | 0 | 4.3 | <MDL | - | <MDL | <MDL | 0 | 4.4 | <MDL | - | <MDL | <MDL | 0 | 4.4 | <MDL | - | <MDL | <MDL | 0 | 47 |
| **CB-95** | <MDL | - | <MDL | <MDL | 0 | 5.7 | 1.3 | 3.4 | <MDL | <MDL - 9.1 | 14 | 4.1 | 9.5 | 25 | <MDL | <MDL - 67 | 14 | 4.1 | <MDL | - | <MDL | <MDL | 0 | 140 |
| **CB-101** | 2.2 | 9.8 | <MDL | <MDL - 61 | 10 | 2.1 | 5.8 | 5.7 | 6.8 | <MDL - 12 | 57 | 4.2 | 25 | 56 | <MDL | <MDL - 150 | 29 | 4.2 | 86 | 230 | <MDL | <MDL - 600 | 14 | 170 |
| **CB-99** | 2.0 | 10 | <MDL | <MDL - 65 | 7.7 | 5.4 | 4.8 | 4.8 | 6.2 | <MDL - 11 | 57 | 4.1 | 16.0 | 31 | <MDL | <MDL - 79 | 43 | 4.1 | <MDL | - | <MDL | <MDL | 0 | 92 |
| **CB-119** | 0.16 | 1.0 | <MDL | <MDL - 6.5 | 2.6 | 2.6 | <MDL | - | <MDL | <MDL | 0 | 5.0 | <MDL | - | <MDL | <MDL | 0 | 5.0 | <MDL | - | <MDL | <MDL | 0 | 54 |
| **CB-87** | <MDL | - | <MDL | <MDL | 0 | 4.3 | <MDL | - | <MDL | <MDL | 0 | 3.0 | 7.2 | 19 | <MDL | <MDL - 50 | 14 | 3.0 | <MDL | - | <MDL | <MDL | 0 | 76 |
| **CB-110** | 0.24 | 1.1 | <MDL | <MDL - 5.5 | 5.1 | 3.4 | 2.4 | 3.1 | <MDL | <MDL - 7.2 | 43 | 2.2 | 11 | 23 | <MDL | <MDL - 62 | 43 | 2.2 | <MDL | - | <MDL | <MDL | 0 | 100 |
| **CB-123** | <MDL | - | <MDL | <MDL | 0 | 6.2 | <MDL | - | <MDL | <MDL | 0 | 6.4 | 3.6 | 10 | <MDL | <MDL - 25 | 14 | 6.4 | <MDL | - | <MDL | <MDL | 0 | 110 |
| **CB-118** | 1.8 | 9.7 | <MDL | <MDL - 61 | 5.1 | 7.8 | 8.8 | 5.4 | 8.4 | <MDL - 18 | 86 | 6.8 | 24 | 56 | <MDL | <MDL - 150 | 43 | 6.8 | <MDL | - | <MDL | <MDL | 0 | 46 |
| **CB-114** | <MDL | - | <MDL | <MDL | 0 | 9.5 | <MDL | - | <MDL | <MDL | 0 | 4.0 | <MDL | - | <MDL | <MDL | 0 | 4.0 | <MDL | - | <MDL | <MDL | 0 | 180 |
| **CB-105** | 0.31 | 2.0 | <MDL | <MDL - 13 | 2.6 | 4.3 | 0.72 | 1.9 | <MDL | <MDL - 5.0 | 29 | 4.8 | 6.1 | 16 | <MDL | <MDL - 43 | 14 | 4.8 | <MDL | - | <MDL | <MDL | 0 | 100 |
| **CB-126** | <MDL | - | <MDL | <MDL | 0 | 5.5 | <MDL | - | <MDL | <MDL | 0 | 4.0 | <MDL | - | <MDL | <MDL | 0 | 4.0 | <MDL | - | <MDL | <MDL | 0 | 89 |
| **CB-155** | 0.44 | 2.8 | <MDL | <MDL - 18 | 2.6 | 3.8 | <MDL | - | <MDL | <MDL | 0 | 3.6 | 1.7 | 4.4 | <MDL | <MDL - 12 | 14 | 3.6 | <MDL | - | <MDL | <MDL | 0 | 110 |
| **CB-151** | 0.76 | 4.9 | <MDL | <MDL - 31 | 2.6 | 6.1 | <MDL | - | <MDL | <MDL | 0 | 4.3 | 8.3 | 22 | <MDL | <MDL - 58 | 14 | 4.3 | <MDL | - | <MDL | <MDL | 0 | 160 |
| **CB-149** | 2.3 | 9.8 | <MDL | <MDL - 61 | 10 | 3.0 | <MDL | - | <MDL | <MDL | 0 | 2.1 | 30 | 61 | <MDL | <MDL - 160 | 29 | 2.1 | <MDL | - | <MDL | <MDL | 0 | 170 |
| **CB-153** | 19 | 53 | 8.0 | <MDL - 340 | 51 | 5.7 | 26 | 7.8 | 24 | 20 - 43 | 100 | 5.6 | 78 | 120 | 12 | <MDL - 310 | 71 | 5.6 | <MDL | - | <MDL | <MDL | 0 | 170 |
| **CB-168** | <MDL | - | <MDL | <MDL | 0 | 3.9 | <MDL | - | <MDL | <MDL | 0 | 3.4 | <MDL | - | <MDL | <MDL | 0 | 3.4 | <MDL | - | <MDL | <MDL | 0 | 96 |
| **CB-138** | 14 | 39 | 3.7 | <MDL - 250 | 49 | 3.5 | 20 | 5.7 | 19 | 15 - 32 | 100 | 3.5 | 52 | 87 | 9.1 | <MDL - 240 | 71 | 3.5 | <MDL | - | <MDL | <MDL | 0 | 130 |
| **CB-158** | 0.35 | 2.3 | <MDL | <MDL - 15 | 2.6 | 5.0 | <MDL | - | <MDL | <MDL | 0 | 3.0 | 2.0 | 5.3 | <MDL | <MDL - 14 | 14 | 3.0 | <MDL | - | <MDL | <MDL | 0 | 110 |
| **CB-128** | 0.68 | 4.4 | <MDL | <MDL - 28 | 2.6 | 4.3 | <MDL | - | <MDL | <MDL | 0 | 10 | 8.5 | 23 | <MDL | <MDL - 60 | 14 | 10 | <MDL | - | <MDL | <MDL | 0 | 120 |

**Table S3.** Concentrations of PCBs in cat sera [pg mL$^{-1}$], dry and wet cat food [pg g$^{-1}$ wet wt.] and house dust [pg g$^{-1}$ dry wt.] (Continued)

| | Cat sera | | | | | | Dry food | | | | | | Wet food | | | | | | House dust | | | | | |
|---|---|---|---|---|---|---|---|---|---|---|---|---|---|---|---|---|---|---|---|---|---|---|---|---|
| **PCBs** | Mean | SD | Median | Range | DF (%) | MDL | Mean | SD | Median | Range | DF (%) | MDL | Mean | SD | Median | Range | DF (%) | MDL | Mean | SD | Median | Range | DF (%) | MDL |
| **CB-167** | 0.20 | 1.2 | <MDL | <MDL - 8.0 | 2.6 | 4.5 | <MDL | - | <MDL | <MDL | 0 | 6.6 | 3.4 | 9.1 | <MDL | <MDL - 24 | 14 | 6.6 | <MDL | - | <MDL | <MDL | 0 | 200 |
| **CB-156** | 0.39 | 2.5 | <MDL | <MDL - 16 | 2.6 | 4.6 | <MDL | - | <MDL | <MDL | 14 | 3.5 | 2.0 | 5.4 | <MDL | <MDL - 14 | 14 | 3.5 | <MDL | - | <MDL | <MDL | 0 | 150 |
| **CB-157** | 0.17 | 1.1 | <MDL | <MDL - 7.0 | 2.6 | 4.6 | <MDL | - | <MDL | <MDL | 0 | 5.9 | <MDL | - | <MDL | <MDL | 0 | 5.9 | <MDL | - | <MDL | <MDL | 0 | 140 |
| **CB-169** | <MDL | - | <MDL | <MDL | 0 | 4.6 | <MDL | - | <MDL | <MDL | 0 | 3.4 | <MDL | - | <MDL | <MDL | 0 | 3.4 | <MDL | - | <MDL | <MDL | 0 | 240 |
| **CB-188** | <MDL | - | <MDL | <MDL | 0 | 4.4 | <MDL | - | <MDL | <MDL | 0 | 5.4 | <MDL | - | <MDL | <MDL | 0 | 5.4 | <MDL | - | <MDL | <MDL | 0 | 76 |
| **CB-178** | 0.58 | 3.7 | <MDL | <MDL - 24 | 2.6 | 5.2 | <MDL | - | <MDL | <MDL | 0 | 5.3 | 2.3 | 6.1 | <MDL | <MDL - 16 | 14 | 5.3 | <MDL | - | <MDL | <MDL | 0 | 120 |
| **CB-187** | 2.5 | 16 | <MDL | <MDL - 100 | 2.6 | 24 | <MDL | - | <MDL | <MDL | 71 | 11 | 27 | 41 | <MDL | <MDL - 96 | 57 | 11 | <MDL | - | <MDL | <MDL | 0 | 110 |
| **CB-183** | 1.1 | 6.9 | <MDL | <MDL - 44 | 2.6 | 18 | <MDL | - | <MDL | <MDL | 14 | 7.7 | 4.2 | 11 | <MDL | <MDL - 29 | 14 | 7.7 | <MDL | - | <MDL | <MDL | 0 | 76 |
| **CB-177** | 0.72 | 4.6 | <MDL | <MDL - 29 | 2.6 | 4.5 | <MDL | - | <MDL | <MDL | 14 | 3.9 | 3.6 | 9.4 | <MDL | <MDL - 25 | 14 | 3.9 | <MDL | - | <MDL | <MDL | 0 | 140 |
| **CB-171** | 0.28 | 1.8 | <MDL | <MDL - 11 | 2.6 | 4.7 | <MDL | - | <MDL | <MDL | 0 | 4.1 | 1.2 | 3.2 | <MDL | <MDL - 8.5 | 14 | 4.1 | <MDL | - | <MDL | <MDL | 0 | 120 |
| **CB-180** | 6.4 | 24 | <MDL | <MDL - 150 | 23 | 2.7 | 13 | 3.3 | 11 | 10 - 20 | 100 | 3.7 | 25 | 31 | 11.0 | <MDL - 81 | 86 | 3.7 | <MDL | - | <MDL | <MDL | 0 | 120 |
| **CB-191** | <MDL | - | <MDL | <MDL | 0 | 4.8 | <MDL | - | <MDL | <MDL | 0 | 4.3 | <MDL | - | <MDL | <MDL | 0 | 4.3 | <MDL | - | <MDL | <MDL | 0 | 73 |
| **CB-170** | 1.5 | 8.5 | <MDL | <MDL - 55 | 5.1 | 2.3 | 0.67 | 1.8 | <MDL | <MDL - 4.7 | 29 | 4.4 | 6.2 | 11 | <MDL | <MDL - 27 | 29 | 4.4 | <MDL | - | <MDL | <MDL | 0 | 120 |
| **CB-189** | 0.18 | 1.1 | <MDL | <MDL - 7.2 | 2.6 | 3.8 | <MDL | - | <MDL | <MDL | 0 | 5.5 | <MDL | - | <MDL | <MDL | 0 | 5.5 | <MDL | - | <MDL | <MDL | 0 | 200 |
| **CB-202** | 0.30 | 1.9 | <MDL | <MDL - 12 | 2.6 | 6.0 | <MDL | - | <MDL | <MDL | 0 | 4.7 | 1.7 | 2.9 | <MDL | <MDL - 6.2 | 29 | 4.7 | <MDL | - | <MDL | <MDL | 0 | 160 |
| **CB-201** | 0.14 | 0.90 | <MDL | <MDL - 5.8 | 2.6 | 2.7 | <MDL | - | <MDL | <MDL | 0 | 7.1 | <MDL | - | <MDL | <MDL | 14 | 7.1 | <MDL | - | <MDL | <MDL | 0 | 140 |
| **CB-199** | 0.70 | 4.5 | <MDL | <MDL - 29 | 2.6 | 5.8 | <MDL | - | <MDL | <MDL | 14 | 6.2 | 3.2 | 6.0 | <MDL | <MDL - 16 | 29 | 6.2 | <MDL | - | <MDL | <MDL | 0 | 200 |
| **CB-194** | 0.86 | 5.5 | <MDL | <MDL - 35 | 2.6 | 7.2 | <MDL | - | <MDL | <MDL | 29 | 4.5 | 3.2 | 5.6 | <MDL | <MDL - 14 | 29 | 4.5 | <MDL | - | <MDL | <MDL | 0 | 160 |
| **CB-205** | <MDL | - | <MDL | <MDL | 0 | 5.0 | <MDL | - | <MDL | <MDL | 0 | 4.6 | <MDL | - | <MDL | <MDL | 0 | 4.6 | <MDL | - | <MDL | <MDL | 0 | 140 |
| **CB-208** | 0.087 | 0.56 | <MDL | <MDL - 3.6 | 2.6 | 3.4 | <MDL | - | <MDL | <MDL | 0 | 2.6 | <MDL | - | <MDL | <MDL | 0 | 2.6 | <MDL | - | <MDL | <MDL | 0 | 100 |
| **CB-206** | 0.21 | 1.3 | <MDL | <MDL - 8.5 | 2.6 | 4.6 | <MDL | - | <MDL | <MDL | 0 | 2.2 | <MDL | - | <MDL | <MDL | 0 | 2.2 | <MDL | - | <MDL | <MDL | 0 | 110 |
| **CB-209** | 0.21 | 1.3 | <MDL | <MDL - 8.4 | 2.6 | 6.7 | <MDL | - | <MDL | <MDL | 0 | 5.5 | 1.5 | 3.9 | <MDL | <MDL - 10 | 14 | 5.5 | <MDL | - | <MDL | <MDL | 0 | 47 |
| **ΣPCBs** | 63 | 240 | 18 | <MDL - 1500 | 74 | | 110 | 53 | 120 | 59 - 210 | 100 | | 410 | 780 | 31 | <MDL - 2100 | 86 | | 110 | 230 | <MDL | <MDL - 600 | 29 | |

**Table S4.** Concentrations of OH-PCBs in cat sera [pg $mL^{-1}$], dry and wet cat food [pg $g^{-1}$ wet wt.] and house dust [pg $g^{-1}$ dry wt.]

| | Cat sera | | | | | | Dry food | | | | | | Wet food | | | | | | House dust | | | | | |
|---|---|---|---|---|---|---|---|---|---|---|---|---|---|---|---|---|---|---|---|---|---|---|---|---|
| OH-PCBs | Mean | SD | Median | Range | DF (%) | MDL | Mean | SD | Median | Range | DF (%) | MDL | Mean | SD | Median | Range | DF (%) | MDL | Mean | SD | Median | Range | DF (%) | MDL |
| **4'OH-CB18** | 21 | 49 | <MDL | <MDL - 310 | 51 | 1.3 | <MDL | - | <MDL | <MDL | 0 | 1.0 | <MDL | - | <MDL | <MDL | 0 | 1.0 | <MDL | - | <MDL | <MDL | 0 | 16 |
| **4'OH-CB35** | <MDL | - | <MDL | <MDL | 0 | 1.3 | <MDL | - | <MDL | <MDL | 0 | 1.0 | <MDL | - | <MDL | <MDL | 0 | 1.0 | <MDL | - | <MDL | <MDL | 0 | 16 |
| **3'OH-CB31** | 6.9 | 44 | <MDL | <MDL - 280 | 2.6 | 1.3 | <MDL | - | <MDL | <MDL | 0 | 1.0 | <MDL | - | <MDL | <MDL | 0 | 1.0 | <MDL | - | <MDL | <MDL | 0 | 16 |
| **3OH-CB25** | <MDL | - | <MDL | <MDL | 0 | 1.3 | <MDL | - | <MDL | <MDL | 0 | 1.0 | <MDL | - | <MDL | <MDL | 0 | 1.0 | <MDL | - | <MDL | <MDL | 0 | 16 |
| **4'OH-CB20** | <MDL | - | <MDL | <MDL | 0 | 1.3 | <MDL | - | <MDL | <MDL | 0 | 1.0 | <MDL | - | <MDL | <MDL | 0 | 1.0 | <MDL | - | <MDL | <MDL | 0 | 16 |
| **3'OH-CB28** | <MDL | - | <MDL | <MDL | 0 | 1.3 | <MDL | - | <MDL | <MDL | 0 | 1.0 | <MDL | - | <MDL | <MDL | 0 | 1.0 | <MDL | - | <MDL | <MDL | 0 | 16 |
| **4OH-CB26** | <MDL | - | <MDL | <MDL | 0 | 1.3 | <MDL | - | <MDL | <MDL | 0 | 1.0 | <MDL | - | <MDL | <MDL | 0 | 1.0 | <MDL | - | <MDL | <MDL | 0 | 16 |
| **4'OH-CB25/26/4OH-CB31** | 16 | 42 | 7 | <MDL - 270 | 82 | 1.3 | <MDL | - | <MDL | <MDL | 0 | 1.0 | <MDL | - | <MDL | <MDL | 0 | 1.0 | <MDL | - | <MDL | <MDL | 0 | 16 |
| **3'OH-CB74** | <MDL | - | <MDL | <MDL | 0 | 5.7 | <MDL | - | <MDL | <MDL | 0 | 1.0 | <MDL | - | <MDL | <MDL | 0 | 1.0 | <MDL | - | <MDL | <MDL | 0 | 150 |
| **4'OH-CB72** | 18 | 15 | 14 | <MDL - 53 | 82 | 5.7 | <MDL | - | <MDL | <MDL | 0 | 1.0 | <MDL | - | <MDL | <MDL | 0 | 1.0 | <MDL | - | <MDL | <MDL | 0 | 150 |
| **3'OH-CB53** | <MDL | - | <MDL | <MDL | 0 | 5.7 | <MDL | - | <MDL | <MDL | 0 | 1.0 | <MDL | - | <MDL | <MDL | 0 | 1.0 | <MDL | - | <MDL | <MDL | 0 | 150 |
| **4'OH-CB65** | <MDL | - | <MDL | <MDL | 0 | 5.7 | <MDL | - | <MDL | <MDL | 0 | 1.0 | <MDL | - | <MDL | <MDL | 0 | 1.0 | <MDL | - | <MDL | <MDL | 0 | 150 |
| **4'OH-CB61** | 3.2 | 6.5 | <MDL | <MDL - 31 | 28 | 5.7 | <MDL | - | <MDL | <MDL | 0 | 1.0 | <MDL | - | <MDL | <MDL | 0 | 1.0 | <MDL | - | <MDL | <MDL | 0 | 150 |
| **4'OH-CB79** | <MDL | - | <MDL | <MDL | 0 | 5.7 | <MDL | - | <MDL | <MDL | 0 | 1.0 | <MDL | - | <MDL | <MDL | 0 | 1.0 | <MDL | - | <MDL | <MDL | 0 | 150 |
| **4OH-CB63** | 0.18 | 1.2 | <MDL | <MDL - 7.6 | 2.6 | 5.7 | <MDL | - | <MDL | <MDL | 0 | 1.0 | <MDL | - | <MDL | <MDL | 0 | 1.0 | <MDL | - | <MDL | <MDL | 0 | 150 |
| **3OH-CB66** | <MDL | - | <MDL | <MDL | 0 | 5.7 | <MDL | - | <MDL | <MDL | 0 | 1.0 | <MDL | - | <MDL | <MDL | 0 | 1.0 | <MDL | - | <MDL | <MDL | 0 | 150 |
| **4OH-CB70** | 14 | 14 | 12 | <MDL - 52 | 69 | 5.7 | <MDL | - | <MDL | <MDL | 0 | 1.0 | <MDL | - | <MDL | <MDL | 0 | 1.0 | <MDL | - | <MDL | <MDL | 0 | 150 |
| **2OH-CB114** | 1.3 | 8.6 | <MDL | <MDL - 55 | 2.6 | 15 | <MDL | - | <MDL | <MDL | 0 | 1.8 | <MDL | - | <MDL | <MDL | 0 | 1.8 | <MDL | - | <MDL | <MDL | 0 | 21 |
| **4OH-CB97** | <MDL | - | <MDL | <MDL | 0 | 15 | <MDL | - | <MDL | <MDL | 0 | 1.8 | <MDL | - | <MDL | <MDL | 0 | 1.8 | <MDL | - | <MDL | <MDL | 0 | 21 |
| **4OH-CB127** | <MDL | - | <MDL | <MDL | 0 | 15 | <MDL | - | <MDL | <MDL | 0 | 1.8 | <MDL | - | <MDL | <MDL | 0 | 1.8 | <MDL | - | <MDL | <MDL | 0 | 21 |
| **3OH-CB118** | 5.7 | 21 | <MDL | <MDL - 130 | 10 | 15 | <MDL | - | <MDL | <MDL | 0 | 1.8 | <MDL | - | <MDL | <MDL | 0 | 1.8 | 14 | 36 | <MDL | <MDL - 95 | 14 | 21 |
| **4'OH-CB106** | <MDL | - | <MDL | <MDL | 0 | 15 | <MDL | - | <MDL | <MDL | 0 | 1.8 | <MDL | - | <MDL | <MDL | 0 | 1.8 | <MDL | - | <MDL | <MDL | 0 | 21 |
| **2OH-CB101** | <MDL | - | <MDL | <MDL | 0 | 15 | <MDL | - | <MDL | <MDL | 0 | 1.8 | <MDL | - | <MDL | <MDL | 0 | 1.8 | <MDL | - | <MDL | <MDL | 0 | 21 |
| **4OH-CB121** | <MDL | - | <MDL | <MDL | 0 | 15 | <MDL | - | <MDL | <MDL | 0 | 1.8 | <MDL | - | <MDL | <MDL | 0 | 1.8 | <MDL | - | <MDL | <MDL | 0 | 21 |
| **3OH-CB101** | 0.51 | 3.3 | <MDL | <MDL - 21 | 2.6 | 15 | <MDL | - | <MDL | <MDL | 0 | 1.8 | <MDL | - | <MDL | <MDL | 0 | 1.8 | <MDL | - | <MDL | <MDL | 0 | 21 |
| **4OH-CB107** | <MDL | - | <MDL | <MDL | 0 | 15 | <MDL | - | <MDL | <MDL | 0 | 1.8 | <MDL | - | <MDL | <MDL | 0 | 1.8 | <MDL | - | <MDL | <MDL | 0 | 21 |
| **4OH-CB108** | <MDL | - | <MDL | <MDL | 0 | 15 | <MDL | - | <MDL | <MDL | 0 | 1.8 | <MDL | - | <MDL | <MDL | 0 | 1.8 | <MDL | - | <MDL | <MDL | 0 | 21 |
| **4OH-CB120** | 0.48 | 3.1 | <MDL | <MDL - 20 | 2.6 | 15 | <MDL | - | <MDL | <MDL | 0 | 1.8 | <MDL | - | <MDL | <MDL | 0 | 1.8 | <MDL | - | <MDL | <MDL | 0 | 21 |
| **4OH-CB101** | 4.4 | 13 | <MDL | <MDL - 79 | 21 | 4.0 | <MDL | - | <MDL | <MDL | 0 | 1.8 | <MDL | - | <MDL | <MDL | 0 | 1.8 | <MDL | - | <MDL | <MDL | 0 | 21 |
| **4OH-CB146** | <MDL | - | <MDL | <MDL | 0 | 4.0 | <MDL | - | <MDL | <MDL | 0 | 1.8 | <MDL | - | <MDL | <MDL | 0 | 1.8 | <MDL | - | <MDL | <MDL | 0 | 60 |
| **4OH-CB130** | <MDL | - | <MDL | <MDL | 0 | 4.0 | <MDL | - | <MDL | <MDL | 0 | 1.8 | <MDL | - | <MDL | <MDL | 0 | 1.8 | <MDL | - | <MDL | <MDL | 0 | 60 |

| | | | | | | | | | | | | | | | | | | | | | | | | |
|---|---|---|---|---|---|---|---|---|---|---|---|---|---|---|---|---|---|---|---|---|---|---|---|---|
| **4OH-CB159** | <MDL | - | <MDL | <MDL | 0 | 4.0 | <MDL | - | <MDL | <MDL | 0 | 1.8 | <MDL | - | <MDL | <MDL | 0 | 1.8 | <MDL | - | <MDL | <MDL | 0 | 60 |
| **4OH-CB162** | 2.3 | 3.9 | <MDL | <MDL - 14 | 31 | 4.0 | <MDL | - | <MDL | <MDL | 0 | 1.8 | <MDL | - | <MDL | <MDL | 0 | 1.8 | <MDL | - | <MDL | <MDL | 0 | 60 |
| **4OH-CB134** | <MDL | - | <MDL | <MDL | 0 | 4.0 | <MDL | - | <MDL | <MDL | 0 | 1.8 | <MDL | - | <MDL | <MDL | 0 | 1.8 | <MDL | - | <MDL | <MDL | 0 | 60 |

**Table S4**. Concentrations of OH-PCBs in cat sera [pg $mL^{-1}$], dry and wet cat food [pg $g^{-1}$ wet wt.] and house dust [pg $g^{-1}$ dry wt.] (Continued)

| | **Cat sera** | | | | | | **Dry food** | | | | | | **Wet food** | | | | | | **House dust** | | | | | |
|---|---|---|---|---|---|---|---|---|---|---|---|---|---|---|---|---|---|---|---|---|---|---|---|---|
| **OH-PCBs** | Mean | SD | Median | Range | DF (%) | MDL | Mean | SD | Median | Range | DF (%) | MDL | Mean | SD | Median | Range | DF (%) | MDL | Mean | SD | Median | Range | DF (%) | MDL |
| **3OH-CB138** | <MDL | - | <MDL | <MDL | 0 | 4.0 | <MDL | - | <MDL | <MDL | 0 | 1.8 | <MDL | - | <MDL | <MDL | 0 | 1.8 | <MDL | - | <MDL | <MDL | 0 | 60 |
| **4OH-CB163** | <MDL | - | <MDL | <MDL | 0 | 4.0 | <MDL | - | <MDL | <MDL | 0 | 1.8 | <MDL | - | <MDL | <MDL | 0 | 1.8 | <MDL | - | <MDL | <MDL | 0 | 60 |
| **4OH-CB165** | 0.29 | 1.9 | <MDL | <MDL - 12 | 2.6 | 4.0 | <MDL | - | <MDL | <MDL | 0 | 1.8 | <MDL | - | <MDL | <MDL | 0 | 1.8 | <MDL | - | <MDL | <MDL | 0 | 60 |
| **3OH-CB153** | 0.24 | 1.5 | <MDL | <MDL - 9.7 | 2.6 | 4.0 | <MDL | - | <MDL | <MDL | 0 | 1.8 | <MDL | - | <MDL | <MDL | 0 | 1.8 | <MDL | - | <MDL | <MDL | 0 | 60 |
| **5OH-CB138** | <MDL | - | <MDL | <MDL | 0 | 4.0 | <MDL | - | <MDL | <MDL | 0 | 1.8 | <MDL | - | <MDL | <MDL | 0 | 1.8 | <MDL | - | <MDL | <MDL | 0 | 60 |
| **3OH-CB184** | <MDL | - | <MDL | <MDL | 0 | 4.0 | <MDL | - | <MDL | <MDL | 0 | 1.8 | <MDL | - | <MDL | <MDL | 0 | 1.8 | <MDL | - | <MDL | <MDL | 0 | 60 |
| **4OH-CB187** | 1.1 | 7.2 | <MDL | <MDL - 46 | 2.6 | 4.0 | <MDL | - | <MDL | <MDL | 0 | 1.8 | <MDL | - | <MDL | <MDL | 0 | 1.8 | <MDL | - | <MDL | <MDL | 0 | 60 |
| **3OH-CB180** | <MDL | - | <MDL | <MDL | 0 | 4.0 | <MDL | - | <MDL | <MDL | 0 | 1.8 | <MDL | - | <MDL | <MDL | 0 | 1.8 | <MDL | - | <MDL | <MDL | 0 | 60 |
| **4OH-CB172** | <MDL | - | <MDL | <MDL | 0 | 4.0 | <MDL | - | <MDL | <MDL | 0 | 1.8 | <MDL | - | <MDL | <MDL | 0 | 1.8 | <MDL | - | <MDL | <MDL | 0 | 60 |
| **4OH-CB193** | <MDL | - | <MDL | <MDL | 0 | 4.0 | <MDL | - | <MDL | <MDL | 0 | 1.8 | <MDL | - | <MDL | <MDL | 0 | 1.8 | <MDL | - | <MDL | <MDL | 0 | 60 |
| **4OH-CB178** | <MDL | - | <MDL | <MDL | 0 | 4.0 | <MDL | - | <MDL | <MDL | 0 | 1.8 | <MDL | - | <MDL | <MDL | 0 | 1.8 | <MDL | - | <MDL | <MDL | 0 | 60 |
| **4OH-CB177** | <MDL | - | <MDL | <MDL | 0 | 4.0 | <MDL | - | <MDL | <MDL | 0 | 1.8 | <MDL | - | <MDL | <MDL | 0 | 1.8 | <MDL | - | <MDL | <MDL | 0 | 60 |
| **5OH-CB183** | 0.72 | 4.6 | <MDL | <MDL - 29 | 0 | 4.0 | <MDL | - | <MDL | <MDL | 0 | 1.8 | <MDL | - | <MDL | <MDL | 0 | 1.8 | <MDL | - | <MDL | <MDL | 0 | 60 |
| **3'OH-CB182/3OH-CB183** | <MDL | - | <MDL | <MDL | 0 | 4.0 | <MDL | - | <MDL | <MDL | 0 | 1.8 | <MDL | - | <MDL | <MDL | 0 | 1.8 | <MDL | - | <MDL | <MDL | 0 | 60 |
| **4OH-CB202** | <MDL | - | <MDL | <MDL | 0 | 4.0 | <MDL | - | <MDL | <MDL | 0 | 1.8 | <MDL | - | <MDL | <MDL | 0 | 1.8 | <MDL | - | <MDL | <MDL | 0 | 60 |
| **4OH-CB199** | <MDL | - | <MDL | <MDL | 0 | 4.0 | <MDL | - | <MDL | <MDL | 0 | 1.8 | <MDL | - | <MDL | <MDL | 0 | 1.8 | <MDL | - | <MDL | <MDL | 0 | 60 |
| **4OH-CB201** | <MDL | - | <MDL | <MDL | 0 | 4.0 | <MDL | - | <MDL | <MDL | 0 | 1.8 | <MDL | - | <MDL | <MDL | 0 | 1.8 | <MDL | - | <MDL | <MDL | 0 | 60 |
| **4OH-CB200** | <MDL | - | <MDL | <MDL | 0 | 4.0 | <MDL | - | <MDL | <MDL | 0 | 1.8 | <MDL | - | <MDL | <MDL | 0 | 1.8 | <MDL | - | <MDL | <MDL | 0 | 60 |
| **3OH-CB203** | <MDL | - | <MDL | <MDL | 0 | 4.0 | <MDL | - | <MDL | <MDL | 0 | 1.8 | <MDL | - | <MDL | <MDL | 0 | 1.8 | <MDL | - | <MDL | <MDL | 0 | 60 |
| **4OH-CB198** | <MDL | - | <MDL | <MDL | 0 | 4.0 | <MDL | - | <MDL | <MDL | 0 | 1.8 | <MDL | - | <MDL | <MDL | 0 | 1.8 | <MDL | - | <MDL | <MDL | 0 | 60 |
| **ΣOH-PCBs** | 97 | 150 | 59 | 1.9 - 1000 | 100 | | <MDL | - | <MDL | <MDL | 0 | | <MDL | - | <MDL | <MDL | 0 | | 14 | 36 | <MDL | <MDL - 95 | 14 | |

**Table S5**. Concentrations of OH-PBDEs in cat sera [pg mL$^{-1}$], dry and wet cat food [pg g$^{-1}$ wet wt.] and house dust [pg g$^{-1}$ dry wt.]

| | Cat sera | | | | | | Dry food | | | | | | Wet food | | | | | | House dust | | | | | |
|---|---|---|---|---|---|---|---|---|---|---|---|---|---|---|---|---|---|---|---|---|---|---|---|---|
| **OH-PBDEs** | Mean | SD | Median | Range | DF (%) | MDL | Mean | SD | Median | Range | DF (%) | MDL | Mean | SD | Median | Range | DF (%) | MDL | Mean | SD | Median | Range | DF (%) | MDL |
| **2'OH-BDE28** | <MDL | - | <MDL | <MDL | 0 | 3.4 | <MDL | - | <MDL | <MDL | 0 | 25 | <MDL | - | <MDL | <MDL | 0 | 25 | <MDL | - | <MDL | <MDL | 0 | 18 |
| **3'OH-BDE28** | <MDL | - | <MDL | <MDL | 0 | 3.4 | <MDL | - | <MDL | <MDL | 0 | 25 | <MDL | - | <MDL | <MDL | 0 | 25 | <MDL | - | <MDL | <MDL | 0 | 18 |
| **4'OH-BDE17** | <MDL | - | <MDL | <MDL | 0 | 3.4 | <MDL | - | <MDL | <MDL | 0 | 25 | <MDL | - | <MDL | <MDL | 0 | 25 | <MDL | - | <MDL | <MDL | 0 | 18 |
| **6'OH-BDE49** | <MDL | - | <MDL | <MDL | 0 | 3.4 | <MDL | - | <MDL | <MDL | 0 | 25 | <MDL | - | <MDL | <MDL | 0 | 25 | <MDL | - | <MDL | <MDL | 0 | 18 |
| **2'OH-BDE68** | 26 | 41 | 16 | <MDL - 190 | 74 | 3.4 | <MDL | - | <MDL | <MDL | 0 | 25 | 17 | 46 | <MDL | <MDL - 120 | 14 | 25 | <MDL | - | <MDL | <MDL | 0 | 18 |
| **6OH-BDE47** | 84 | 82 | 55 | 9.9 - 370 | 100 | 3.4 | <MDL | - | <MDL | <MDL | 0 | 8.2 | 6.7 | 13 | <MDL | <MDL - 35 | 29 | 8.2 | <MDL | - | <MDL | <MDL | 0 | 12 |
| **3OH-BDE47** | <MDL | - | <MDL | <MDL | 0 | 3.4 | <MDL | - | <MDL | <MDL | 0 | 8.2 | <MDL | - | <MDL | <MDL | 0 | 8.2 | <MDL | - | <MDL | <MDL | 0 | 12 |
| **5OH-BDE47** | <MDL | - | <MDL | <MDL | 0 | 3.4 | <MDL | - | <MDL | <MDL | 0 | 8.2 | <MDL | - | <MDL | <MDL | 0 | 8.2 | <MDL | - | <MDL | <MDL | 0 | 12 |
| **4OH-BDE49** | <MDL | - | <MDL | <MDL | 0 | 3.4 | <MDL | - | <MDL | <MDL | 0 | 8.2 | <MDL | - | <MDL | <MDL | 0 | 8.2 | <MDL | - | <MDL | <MDL | 0 | 12 |
| **4'OH-BDE42** | <MDL | - | <MDL | <MDL | 0 | 3.4 | <MDL | - | <MDL | <MDL | 0 | 8.2 | <MDL | - | <MDL | <MDL | 0 | 8.2 | <MDL | - | <MDL | <MDL | 0 | 12 |
| **5'OH-BDE100** | <MDL | - | <MDL | <MDL | 0 | 3.4 | <MDL | - | <MDL | <MDL | 0 | 8.2 | <MDL | - | <MDL | <MDL | 0 | 8.2 | <MDL | - | <MDL | <MDL | 0 | 12 |
| **4'OH-BDE103** | <MDL | - | <MDL | <MDL | 0 | 3.4 | <MDL | - | <MDL | <MDL | 0 | 8.2 | <MDL | - | <MDL | <MDL | 0 | 8.2 | <MDL | - | <MDL | <MDL | 0 | 12 |
| **6OH-BDE99** | <MDL | - | <MDL | <MDL | 0 | 3.4 | <MDL | - | <MDL | <MDL | 0 | 8.2 | <MDL | - | <MDL | <MDL | 0 | 8.2 | <MDL | - | <MDL | <MDL | 0 | 12 |
| **5'OH-BDE99** | <MDL | - | <MDL | <MDL | 0 | 3.4 | <MDL | - | <MDL | <MDL | 0 | 8.2 | <MDL | - | <MDL | <MDL | 0 | 8.2 | <MDL | - | <MDL | <MDL | 0 | 12 |
| **4OH-BDE90** | <MDL | - | <MDL | <MDL | 0 | 3.4 | <MDL | - | <MDL | <MDL | 0 | 8.2 | <MDL | - | <MDL | <MDL | 0 | 8.2 | <MDL | - | <MDL | <MDL | 0 | 12 |
| **4'OH-BDE101** | <MDL | - | <MDL | <MDL | 0 | 3.4 | <MDL | - | <MDL | <MDL | 0 | 8.2 | <MDL | - | <MDL | <MDL | 0 | 8.2 | <MDL | - | <MDL | <MDL | 0 | 12 |
| **ΣOH-PBDEs** | 110 | 100 | 77 | 9.9 - 440 | 100 | | <MDL | - | <MDL | <MDL | 0 | | 24 | 59 | <MDL | <MDL - 160 | 29 | | <MDL | - | <MDL | <MDL | 0 | |

**Table S6.** Concentrations of MeO-PBDEs in cat sera [pg $mL^{-1}$], dry and wet cat food [pg $g^{-1}$ wet wt.] and house dust [pg $g^{-1}$ dry wt.]

| | Cat sera | | | | | | Dry food | | | | | | Wet food | | | | | | House dust | | | | | |
|---|---|---|---|---|---|---|---|---|---|---|---|---|---|---|---|---|---|---|---|---|---|---|---|---|
| **MeO-PBDEs** | Mean | SD | Median | Range | DF (%) | MDL | Mean | SD | Median | Range | DF (%) | MDL | Mean | SD | Median | Range | DF (%) | MDL | Mean | SD | Median | Range | DF (%) | MDL |
| **2'MeO-BDE28** | <MDL | <MDL | <MDL | <MDL | 0 | 3.4 | <MDL | - | <MDL | <MDL | 0 | 25 | <MDL | - | <MDL | <MDL | 0 | 25 | <MDL | - | <MDL | <MDL | 0 | 290 |
| **3'MeO-BDE28** | <MDL | <MDL | <MDL | <MDL | 0 | 3.4 | <MDL | - | <MDL | <MDL | 0 | 25 | <MDL | - | <MDL | <MDL | 0 | 25 | <MDL | - | <MDL | <MDL | 0 | 290 |
| **4'MeO-BDE17** | <MDL | <MDL | <MDL | <MDL | 0 | 3.4 | <MDL | - | <MDL | <MDL | 0 | 25 | <MDL | - | <MDL | <MDL | 0 | 25 | <MDL | - | <MDL | <MDL | 0 | 290 |
| **6'MeO-BDE49** | <MDL | <MDL | <MDL | <MDL | 0 | 3.4 | <MDL | - | <MDL | <MDL | 0 | 25 | <MDL | - | <MDL | <MDL | 0 | 25 | <MDL | - | <MDL | <MDL | 0 | 290 |
| **2'MeO-BDE68** | 22 | 29 | 14 | <MDL - 130 | 59 | 3.4 | <MDL | - | <MDL | <MDL | 0 | 25 | 490 | 1000 | 71 | <MDL - 2800 | 71 | 25 | <MDL | - | <MDL | <MDL | 0 | 290 |
| **6MeO-BDE47** | 27 | 68 | <MDL | <MDL - 380 | 28 | 3.4 | 12 | 12 | 18 | <MDL - 25 | 57 | 8.2 | 83 | 160 | 17 | <MDL - 420 | 57 | 8.2 | <MDL | - | <MDL | <MDL | 0 | 98 |
| **3MeO-BDE47** | <MDL | <MDL | <MDL | <MDL | 0 | 3.4 | <MDL | - | <MDL | <MDL | 0 | 8.2 | <MDL | - | <MDL | <MDL | 0 | 8.2 | <MDL | - | <MDL | <MDL | 0 | 98 |
| **5MeO-BDE47** | <MDL | <MDL | <MDL | <MDL | 0 | 3.4 | <MDL | - | <MDL | <MDL | 0 | 8.2 | <MDL | - | <MDL | <MDL | 0 | 8.2 | <MDL | - | <MDL | <MDL | 0 | 98 |
| **4MeO-BDE49** | <MDL | <MDL | <MDL | <MDL | 0 | 3.4 | <MDL | - | <MDL | <MDL | 0 | 8.2 | <MDL | - | <MDL | <MDL | 0 | 8.2 | <MDL | - | <MDL | <MDL | 0 | 98 |
| **4'MeO-BDE42** | <MDL | <MDL | <MDL | <MDL | 0 | 3.4 | <MDL | - | <MDL | <MDL | 0 | 8.2 | <MDL | - | <MDL | <MDL | 0 | 8.2 | <MDL | - | <MDL | <MDL | 0 | 98 |
| **5'MeO-BDE100** | <MDL | <MDL | <MDL | <MDL | 0 | 3.4 | <MDL | - | <MDL | <MDL | 0 | 8.2 | <MDL | - | <MDL | <MDL | 0 | 8.2 | <MDL | - | <MDL | <MDL | 0 | 98 |
| **4'MeO-BDE103** | <MDL | <MDL | <MDL | <MDL | 0 | 3.4 | <MDL | - | <MDL | <MDL | 0 | 8.2 | <MDL | - | <MDL | <MDL | 0 | 8.2 | <MDL | - | <MDL | <MDL | 0 | 98 |
| **6MeO-BDE99** | 0.30 | 1.9 | <MDL | <MDL - 12 | 2.6 | 3.4 | <MDL | - | <MDL | <MDL | 0 | 8.2 | <MDL | - | <MDL | <MDL | 0 | 8.2 | <MDL | - | <MDL | <MDL | 0 | 98 |
| **5'MeO-BDE99** | <MDL | <MDL | <MDL | <MDL | 0 | 3.4 | <MDL | - | <MDL | <MDL | 0 | 8.2 | <MDL | - | <MDL | <MDL | 0 | 8.2 | <MDL | - | <MDL | <MDL | 0 | 98 |
| **4MeO-BDE90** | <MDL | <MDL | <MDL | <MDL | 0 | 3.4 | <MDL | - | <MDL | <MDL | 0 | 8.2 | <MDL | - | <MDL | <MDL | 0 | 8.2 | <MDL | - | <MDL | <MDL | 0 | 98 |
| **4'MeO-BDE101** | <MDL | <MDL | <MDL | <MDL | 0 | 3.4 | <MDL | - | <MDL | <MDL | 0 | 8.2 | <MDL | - | <MDL | <MDL | 0 | 8.2 | <MDL | - | <MDL | <MDL | 0 | 98 |
| **ΣMeO-PBDEs** | 49 | 82 | 19 | <MDL - 460 | 62 | | 12 | 12 | 18 | <MDL - 25 | 57 | | 520 | 1200 | <MDL | <MDL - 3300 | 29 | | <MDL | - | <MDL | <MDL | 0 | |

**Table S7.** Lipid-based concentrations (ng $g^{-1}$) of OHCs in cat serum ($n$ = 26)

| Compound | Median (Range) | DF (%) |
|---|---|---|
| BDE-47 | <MDL (<MDL-47) | 38 |
| BDE-99 | <MDL (<MDL-64) | 33 |
| BDE-209 | 170 (<MDL-16000) | 92 |
| **ΣPBDEs** | **230 (<MDL-21000)** | **96** |
| CB-153 | 3.7 (<MDL-230) | 58 |
| CB-138 | 1.3 (<MDL-170) | 50 |
| **ΣPCBs** | **10 (<MDL-1000)** | **79** |
| 4'-OH-CB-25/26/4-OH-CB-31 | 3 (<MDL-150) | 88 |
| 4'-OH-CB-72 | 5.8 (<MDL-38) | 88 |
| **ΣOH-PCBs** | **23 (0.79-550)** | **100** |
| 2'-OH-BDE-68 | 7.7 (<MDL-120) | 83 |
| 6-OH-BDE-47 | 22 (4.3-160) | 100 |
| **ΣOH-PBDEs** | **27 (7.8-270)** | **100** |
| 2'-MeO-BDE-68 | 6.8 (<MDL-87) | 79 |
| 6-MeO-BDE-47 | <MDL (<MDL-160) | 46 |
| **ΣMeO-PBDEs** | **12 (<MDL-190)** | **79** |

**Table S8.** Correlations between the concentrations [wet wt. base] of OHCs in cat sera and the results of questionnaires

| | | PBDEs | BDE-47 | BDE-99 | BDE-209 | PCBs | OH-PCBs | OH-PBDEs | MeO-PBDEs | Age [year] | Weight [kg] | Ratio A | Ratio B |
|---|---|---|---|---|---|---|---|---|---|---|---|---|---|
| PBDEs | Correlation coefficient | | 0.096 | 0.268 | **0.850** | -0.057 | 0.118 | 0.196 | 0.093 | -0.045 | -0.129 | -0.007 | 0.109 |
| | *p*-value | | 0.549 | 0.090 | **0.000** | 0.726 | 0.459 | 0.219 | 0.563 | 0.782 | 0.421 | 0.967 | 0.496 |
| BDE-47 | Correlation coefficient | 0.096 | | **0.717** | 0.134 | **0.717** | 0.303 | 0.278 | 0.305 | **0.357** | 0.030 | -0.060 | 0.194 |
| | *p*-value | 0.549 | | **0.000** | 0.405 | **0.000** | 0.054 | 0.078 | 0.053 | **0.022** | 0.853 | 0.710 | 0.224 |
| BDE-99 | Correlation coefficient | 0.268 | **0.717** | | 0.285 | **0.530** | **0.326** | 0.170 | 0.126 | 0.213 | -0.109 | 0.072 | -0.099 |
| | *p*-value | 0.090 | **0.000** | | 0.071 | **0.000** | **0.038** | 0.287 | 0.434 | 0.181 | 0.496 | 0.655 | 0.540 |
| BDE-209 | Correlation coefficient | **0.850** | 0.134 | 0.285 | | -0.089 | 0.047 | 0.291 | 0.257 | 0.070 | -0.069 | 0.009 | 0.139 |
| | *p*-value | **0.000** | 0.405 | 0.071 | | 0.580 | 0.769 | 0.065 | 0.105 | 0.666 | 0.668 | 0.956 | 0.385 |
| PCBs | Correlation coefficient | -0.057 | **0.717** | **0.530** | -0.089 | | **0.337** | 0.190 | **0.331** | **0.352** | 0.026 | -0.036 | 0.022 |
| | *p*-value | 0.726 | **0.000** | **0.000** | 0.580 | | **0.031** | 0.233 | **0.035** | **0.024** | 0.873 | 0.822 | 0.892 |
| OH-PCBs | Correlation coefficient | 0.118 | 0.303 | **0.326** | 0.047 | **0.337** | | **0.333** | -0.055 | 0.286 | -0.054 | -0.212 | 0.146 |
| | *p*-value | 0.459 | 0.054 | **0.038** | 0.769 | **0.031** | | **0.034** | 0.733 | 0.070 | 0.739 | 0.184 | 0.363 |
| OH-PBDEs | Correlation coefficient | 0.196 | 0.278 | 0.170 | 0.291 | 0.190 | **0.333** | | **0.364** | 0.120 | 0.114 | -0.280 | 0.252 |
| | *p*-value | 0.219 | 0.078 | 0.287 | 0.065 | 0.233 | **0.034** | | **0.019** | 0.456 | 0.476 | 0.076 | 0.112 |
| MeO-PBDEs | Correlation coefficient | 0.093 | 0.305 | 0.126 | 0.257 | **0.331** | -0.055 | **0.364** | | 0.123 | **0.317** | 0.052 | 0.109 |
| | *p*-value | 0.563 | 0.053 | 0.434 | 0.105 | **0.035** | 0.733 | **0.019** | | 0.443 | **0.043** | 0.745 | 0.499 |
| Age [year] | Correlation coefficient | -0.045 | **0.357** | 0.213 | 0.070 | **0.352** | 0.286 | 0.120 | 0.123 | | **0.453** | -0.185 | **0.457** |
| | *p*-value | 0.782 | **0.022** | 0.181 | 0.666 | **0.024** | 0.070 | 0.456 | 0.443 | | **0.003** | 0.248 | **0.003** |
| Weight [kg] | Correlation coefficient | -0.129 | 0.030 | -0.109 | -0.069 | 0.026 | -0.054 | 0.114 | **0.317** | **0.453** | | -0.129 | **0.364** |
| | *p*-value | 0.421 | 0.853 | 0.496 | 0.668 | 0.873 | 0.739 | 0.476 | **0.043** | **0.003** | | 0.420 | **0.019** |
| Ratio A* | Correlation coefficient | -0.007 | -0.060 | 0.072 | 0.009 | -0.036 | -0.212 | -0.280 | 0.052 | -0.185 | -0.129 | | **-0.749** |
| | *p*-value | 0.967 | 0.710 | 0.655 | 0.956 | 0.822 | 0.184 | 0.076 | 0.745 | 0.248 | 0.420 | | **0.000** |
| Ratio B** | Correlation coefficient | 0.109 | 0.194 | -0.099 | 0.139 | 0.022 | 0.146 | 0.252 | 0.109 | **0.457** | **0.364** | **-0.749** | |
| | *p*-value | 0.496 | 0.224 | 0.540 | 0.385 | 0.892 | 0.363 | 0.112 | 0.499 | **0.003** | **0.019** | **0.000** | |

*Ratio A was calculated by weekly feed number of times of dry food divided by total feed number of times.

**Ratio B was calculated by weekly feed number of times of wet food divided by total feed number of times.

**Table S9.** Correlations between the concentrations [lipid wt. base] of OHCs in cat sera and the results of questionnaires[*]

| | | PBDEs | BDE-47 | BDE-99 | BDE-209 | PCBs | OH-PCBs | OH-PBDEs | MeO-PBDEs | Age [year] | Weight [kg] | Lipid | Ratio A | Ratio B |
|---|---|---|---|---|---|---|---|---|---|---|---|---|---|---|
| PBDEs | Correlation coefficient | | 0.301 | 0.305 | 0.201 | -0.045 | 0.356 | **0.481** | 0.128 | -0.263 | **-0.459** | **-0.456** | -0.081 | -0.126 |
| | *p*-value | | 0.153 | 0.148 | 0.347 | 0.836 | 0.089 | 0.018 | 0.550 | 0.214 | **0.025** | 0.026 | 0.707 | 0.557 |
| BDE-47 | Correlation coefficient | 0.301 | | **0.767** | 0.061 | 0.158 | 0.301 | 0.164 | 0.108 | 0.059 | -0.210 | -0.201 | 0.166 | -0.208 |
| | *p*-value | 0.153 | | **0.000** | 0.777 | 0.462 | 0.153 | 0.444 | 0.614 | 0.785 | 0.325 | 0.346 | 0.439 | 0.329 |
| BDE-99 | Correlation coefficient | 0.305 | **0.767** | | 0.320 | 0.069 | 0.389 | 0.227 | 0.179 | 0.187 | -0.060 | **-0.411** | 0.203 | -0.079 |
| | *p*-value | 0.148 | **0.000** | | 0.128 | 0.748 | 0.060 | 0.286 | 0.403 | 0.383 | 0.782 | **0.046** | 0.342 | 0.713 |
| BDE-209 | Correlation coefficient | 0.201 | 0.061 | 0.320 | | -0.080 | **0.463** | 0.144 | -0.166 | 0.127 | -0.044 | **-0.564** | 0.013 | -0.021 |
| | *p*-value | 0.347 | 0.777 | 0.128 | | 0.709 | **0.023** | 0.502 | 0.438 | 0.553 | 0.839 | **0.004** | 0.952 | 0.921 |
| PCBs | Correlation coefficient | -0.045 | 0.158 | 0.069 | -0.080 | | **0.400** | 0.240 | 0.165 | 0.209 | -0.002 | -0.122 | -0.390 | -0.174 |
| | *p*-value | 0.836 | 0.462 | 0.748 | 0.709 | | 0.053 | 0.258 | 0.441 | 0.327 | 0.994 | 0.569 | 0.060 | 0.416 |
| OH-PCBs | Correlation coefficient | 0.356 | 0.301 | 0.389 | **0.463** | **0.400** | | **0.569** | 0.029 | 0.209 | -0.386 | **-0.657** | **-0.460** | -0.013 |
| | *p*-value | 0.089 | 0.153 | 0.060 | **0.023** | **0.053** | | **0.004** | 0.894 | 0.326 | 0.063 | **0.001** | **0.024** | 0.953 |
| OH-PBDEs | Correlation coefficient | **0.481** | 0.164 | 0.227 | 0.144 | 0.240 | **0.569** | | 0.324 | 0.164 | -0.108 | **-0.684** | -0.227 | 0.212 |
| | *p*-value | **0.018** | 0.444 | 0.286 | 0.502 | 0.258 | **0.004** | | 0.122 | 0.445 | 0.615 | **0.000** | 0.287 | 0.321 |
| MeO-PBDEs | Correlation coefficient | 0.128 | 0.108 | 0.179 | -0.166 | 0.165 | 0.029 | 0.324 | | 0.016 | 0.118 | -0.002 | -0.007 | 0.096 |
| | *p*-value | 0.550 | 0.614 | 0.403 | 0.438 | 0.441 | 0.894 | 0.122 | | 0.940 | 0.583 | 0.994 | 0.973 | 0.656 |
| Age [year] | Correlation coefficient | -0.263 | 0.059 | 0.187 | 0.127 | 0.209 | 0.209 | 0.164 | 0.016 | | **0.543** | -0.305 | 0.112 | 0.345 |
| | *p*-value | 0.214 | 0.785 | 0.383 | 0.553 | 0.327 | 0.326 | 0.445 | 0.940 | | **0.006** | 0.148 | 0.602 | 0.099 |
| Weight [kg] | Correlation coefficient | **-0.459** | -0.210 | -0.060 | -0.044 | -0.002 | -0.386 | -0.108 | 0.118 | **0.543** | | 0.235 | **0.440** | **0.431** |
| | *p*-value | **0.025** | 0.325 | 0.782 | 0.839 | 0.994 | 0.063 | 0.615 | 0.583 | **0.006** | | 0.268 | **0.032** | **0.035** |
| Living | Correlation coefficient | **-0.456** | -0.201 | **-0.411** | **-0.564** | -0.122 | **-0.657** | **-0.684** | -0.002 | -0.305 | 0.235 | | 0.195 | -0.001 |
| | *p*-value | **0.026** | 0.346 | **0.046** | **0.004** | 0.569 | **0.001** | **0.000** | 0.994 | 0.148 | 0.268 | | 0.360 | 0.997 |
| Ratio A | Correlation coefficient | -0.081 | 0.166 | 0.203 | 0.013 | -0.390 | **-0.460** | -0.227 | -0.007 | 0.112 | **0.440** | 0.195 | | 0.235 |
| | *p*-value | 0.707 | 0.439 | 0.342 | 0.952 | 0.060 | **0.024** | 0.287 | 0.973 | 0.602 | **0.032** | 0.360 | | 0.269 |
| Ratio B | Correlation coefficient | -0.126 | -0.208 | -0.079 | -0.021 | -0.174 | -0.013 | 0.212 | 0.096 | 0.345 | **0.431** | -0.001 | 0.235 | |
| | *p*-value | 0.557 | 0.329 | 0.713 | 0.921 | 0.416 | 0.953 | 0.321 | 0.656 | 0.099 | **0.035** | 0.997 | 0.269 | |

*Ratio A was calculated by weekly feed number of times of dry food divided by total feed number of times.

**Ratio B was calculated by weekly feed number of times of wet food divided by total feed number of times.

**Table S10.** Estimation of daily intake (DI) and hazard quotient (HQ) of BDE-47 via cat food and house dust.

| Source | | Concentration (pg/g) | Intake of pet food (g) | Intake of house dust (mg) | DI (ng/kg/day) |
|---|---|---|---|---|---|
| **Dry food** | median | 4.9 | 78 [b] | - | 0.1 |
| | worst | 15 | 120 [c] | - | 0.5 |
| **Dry and Wet food** [a] | median | - | 230 [b] | - | 0.3 |
| | worst | - | 270 [c] | - | 2.6 |
| **Wet food** | median | 5.6 | 380 [b] | - | 0.5 |
| | worst | 46 | 420 [c] | - | 4.8 |
| **House dust** | median | 260 | - | 50 [d] | 0.0033 |
| | worst | 890 | - | 200 [e] | 0.045 |

[a] Half of dry food and wet food

[b] Average of recommended feed amount for 4 kg cat written in package labels.

[c] Maximum of recommended feed amount for 4 kg cat written in package labels.

[d] Extrapolated the standard value of house dust intake to infants (Dirtu et al., 2010)

[e] Extrapolated the maximum of house dust intake to infants (Dirtu et al., 2010)

**Table. S11.** Estimation of daily intake (DI) of BDE-99 via cat food and house dust.

| Source | | Concentration (pg/g) | Intake of pet food (g) | Intake of house dust (mg) | DI (ng/kg/day) |
|---|---|---|---|---|---|
| **Dry food** | median | <MDL | 78 [b] | - | 0.0 |
| | worst | 12 | 120 [c] | - | 0.4 |
| **Dry and Wet food** [a] | median | - | 230 [b] | - | 0.0 |
| | worst | - | 270 [c] | - | 0.8 |
| **Wet food** | median | <MDL | 380 [b] | - | 0.0 |
| | worst | 11 | 420 [c] | - | 1.2 |
| **House dust** | median | 210 | - | 50 [d] | 0.0026 |
| | worst | 1400 | - | 200 [e] | 0.070 |

[a] Half of dry food and wet food

[b] Average of recommended feed amount for 4 kg cat written in package labels.

[c] Maximum of recommended feed amount for 4 kg cat written in package labels.

[d] Extrapolated the standard value of house dust intake to infants (Dirtu et al., 2010)

[e] Extrapolated the maximum of house dust intake to infants (Dirtu et al., 2010

**Table S12.** Estimation of daily intake (DI) of BDE-153 via cat food and house dust.

| **Source** | | **Concentration (pg/g)** | **Intake of pet food (g)** | **Intake of house dust (mg)** | **DI (ng/kg/day)** |
|---|---|---|---|---|---|
| **Dry food** | median | <MDL | 78 [b] | - | 0.0 |
| | worst | <MDL | 120 [c] | - | 0.0 |
| **Dry and Wet food** [a] | median | - | 230 [b] | - | 0.0 |
| | worst | - | 270 [c] | - | 0.0 |
| **Wet food** | median | <MDL | 380 [b] | - | 0.0 |
| | worst | <MDL | 420 [c] | - | 0.0 |
| **House dust** | median | <MDL | - | 50 [d] | 0.0 |
| | worst | 430 | - | 200 [e] | 0.022 |

[a] Half of dry food and wet food

[b] Average of recommended feed amount for 4 kg cat written in package labels.

[c] Maximum of recommended feed amount for 4 kg cat written in package labels.

[d] Extrapolated the standard value of house dust intake to infants (Dirtu et al., 2010)

[e] Extrapolated the maximum of house dust intake to infants (Dirtu et al., 2010)

**Table S13.** Estimation of daily intake (DI) of BDE-209 via cat food and house dust.

| Source | | Concentration (pg/g) | Intake of cat food (g) | Intake of house dust (mg) | DI (ng/kg/day) |
|---|---|---|---|---|---|
| **Dry food** | median | 330 | 78 [b] | - | 6.4 |
| | worst | 31000 | 120 [c] | - | 930 |
| **Dry and Wet food [a]** | median | - | 230 [b] | - | 3.8 |
| | worst | - | 270 [c] | - | 475 |
| **Wet food** | median | 12 | 380 [b] | - | 1.1 |
| | worst | 190 | 420 [c] | - | 20 |
| **House dust** | median | 200000 | - | 50 [d] | 2.5 |
| | worst | 520000 | - | 200 [e] | 26 |

[a] Half of dry food and wet food

[b] Average of recommended feed amount for 4 kg cat written in package labels.

[c] Maximum of recommended feed amount for 4 kg cat written in package labels.

[d] Extrapolated the standard value of house dust intake to infants (Dirtu et al., 2010)

[e] Extrapolated the maximum of house dust intake to infants (Dirtu et al., 2010)
